# Plastome of *Quercus xanthoclada* and comparison of genomic diversity amongst selected *Quercus* species using genome skimming

**DOI:** 10.3897/phytokeys.132.36365

**Published:** 2019-10-01

**Authors:** Damien Daniel Hinsinger, Joeri Sergej Strijk

**Affiliations:** 1 Biodiversity Genomics Team, Plant Ecophysiology & Evolution Group, Guangxi Key Laboratory of Forest Ecology and Conservation, College of Forestry, Daxuedonglu 100, Nanning, Guangxi, 530005, China; 2 State Key Laboratory for Conservation and Utilization of Subtropical Agro-bioresources, College of Forestry, Guangxi University, Nanning, Guangxi 530005, China; 3 Alliance for Conservation Tree Genomics, Pha Tad Ke Botanical Garden, PO Box 959, 06000 Luang Prabang, Lao PDR

**Keywords:** Phylogenomics, Quercus, Plastid genome, Genomic Diversity, Diversification, Sections, Taxonomy, Fagaceae, SNPs, Genomic Resources

## Abstract

The genus *Quercus* L. contains several of the most economically important species for timber production in the Northern Hemisphere. It was one of the first genera described, but genetic diversity at a global scale within and amongst oak species remains unclear, despite numerous regional or species-specific assessments. To evaluate global plastid diversity in oaks, we sequenced the complete chloroplast of *Quercus xanthoclada* and compared its sequence with those available from other main taxonomic groups in *Quercus*. We quantify genomic divergence amongst oaks and performed a sliding window analysis to detect the most variable regions amongst members of the various clades, as well as divergent regions occurring in specific pairs of species. We identified private and shared SNPs amongst oaks species and sections and stress the need for a large global assessment of genetic diversity in this economically and ecologically important genus.

## Introduction

The genus *Quercus* in Fagaceae, a large and locally dominant family of trees in temperate forests of the Northern Hemisphere and in major regions of the tropics and subtropics, is one of the first plant genera described in history (Linnaeus 1753). Fagaceae comprise 8 well-recognised genera, holding ±900–1100 economically and ecologically important species (Manos et al. 2001). In the family, *Quercus* is the largest genus, with ±500–550 accepted species of broad-leaved, deciduous or evergreen trees and shrubs (Denk et al. 2017; Nixon 2006). Although best known from Europe and North America, it extends well into Central and South America, as well as the Asian tropical regions (Indo-China, Malesia) with large numbers of species (Soepadmo and Steenis 1972; Pulido et al. 2006; Phengklai 2008). South America appears to one of the most recent areas to be colonized by the genus, with *Q. humboldtii* occurring as far south as Colombia (Pulido et al. 2006). Until recently, the genus was divided in five subgenera and sections, based on molecular (Oh and Manos 2008), pollen morphology (Denk and Grimm 2009), morphological (Luo and Zhou 2002) and historical treatments (Camus 1936–1954). This was changed with a major update of the infrageneric classification (Denk et al. 2017). The genus is now divided into two subgenera holding a total of 8 sections (subg. Quercus: *Protobalanus*, *Ponticae*, *Virentes*, *Quercus*, Lobatae ; and subg. Cerris: *Ilex*, *Cyclobalanopsis*, *Cerris*) (Denk et al. 2017). China, where oaks are among the main components of southern broad-leaved evergreen forests, holds the highest number of Asian species (Huang et al. 2009; Strijk 2019). *Quercus* and other Fagaceae are often the dominant element in tropical and sub-tropical evergreen forests (Huang et al. 1998) and, like Dipterocarpaceae in Asia, repre-sent an exceptional ecological and economical resource (Cvetković et al. 2017; 2019).

Chloroplast DNA loci have been widely used in plant studies, both for evolutionary studies and for identification purposes, due to their natural abundance in plant cells (≈3–5% of the cell DNA content), when compared to nuclear DNA. In angiosperms, the chloroplast genome is a circular molecule (76–217 kilobases), with a conserved structure of two inverted repeats (IR) separated by small (SSC) and large (LSC) single-copy regions (Jansen and Ruhlman 2012). However, until recently, the number of regions in the chloroplast genome, used to address evolutionary, taxonomic and biodiversity questions, remained limited. With the rapid development of Next Generation Sequencing (NGS) approaches, analysing the entire sequence of the chloroplast using a genome skimming approach (Straub et al. 2012) has become common practice and efficient, allowing for high resolution phylogenies (Ripma et al. 2014), resolved problematic taxonomic placements (Bock et al. 2014; Zedane et al. 2016), and estimates of genomic biodiversity (Särkinen et al. 2012; Staats et al. 2013; Bakker et al. 2016). Although geography can be a major factor in shaping plastome diversity at small scales (Pham et al. 2017), dominant patterns of genomic diversity are shaped by evolutionary processes. In oaks, plastome sequences of about 20 species have been studied and are currently available online (sometimes with numerous conspecific accessions, of which many lack essential voucher information), including *Q. rubra* (Alexander and Woeste 2014), *Q. spinosa* (Du et al. 2015), *Q. aquifolioides* and *Q. aliena* (Lu et al. 2016). These sequences can thus be used in reference-guided assemblies (Ripma et al. 2014) and applied to assess the chloroplast diversity in the genus. These species – in addition to the *Q. xanthoclada* chloroplast sequence we generated – belong to the four most diverse lineages (representing more than 97% of species-level diversity). The main aim of our study was to assess levels of genetic diversity contained within the chloroplast, using a subset of oaks from key clades and using a genome skimming approach to reconstruct the complete sequence of the chloroplast for *Q. xanthoclada*. Using *xanthoclada*, we performed phylogenetic and sliding window analyses, single-nucleotide polymorphisms (SNPs) detection and genomic comparisons of diversity.

## Materials and methods

### Chloroplast reconstruction

Genomic DNA was extracted from 0.1 g of silica gel-dehydrated leaves using a protocol modified from Healey et al. (2014). Modifications were as follows: genomic DNA was extracted in 15 ml tubes, using 6 ml of extraction buffer, incubated at 65 °C for 60 min and two volumes of temperate absolute ethanol were added for precipitation, without incubation. Library construction and sequencing were performed by Novogene (Beijing, China), using NEBNext Ultra II DNA Library Prep Kit (Ipswich, Massachusetts, USA) and an Illumina HiSeq2500 platform (San Diego, California, USA), respectively, following specifications from the manufacturer. One Gb of raw data was generated and was imported in Geneious R11 (Biomatters Ltd, Auckland, New Zealand). Raw reads were trimmed according to their 5’ and 3’-end quality and then mapped against the available chloroplast of *Q. rubra* (NC020152). Annotations were made using cpGAVAS (Liu et al. 2012) and boundary validation was performed, using ORF Finder (NCBI), with manual adjustments. The complete plastid genome was submitted to GenBank under accession number KU382355. The GenBank flatfile was used to generate a circular plastid genome map using the OrganellarGenomeDraw (OGDRAW) (Lohse et al. 2007).

### Phylogenetic reconstruction

Following the recommended best practices for complete organellar sequencing (Botero-Castro et al. 2016), we performed a phylogenetic analysis to confirm the accuracy of our reconstructed plastid and our sample identification. We retrieved all the complete chloroplasts available in GenBank for the Fagales order (accessed 25 January 2019) and built both maximum likelihood and Bayesian trees using the PHYML (Guindon et al. 2009) implementation in Geneious R11 and BEAST 2.3.1 (Bouckaert et al. 2014), respectively. The ML tree was built using all positions, aligned using MAFFT (Katoh and Standley 2013) with default parameters and bootstrap values were calculated using 1000 replicates. We conducted BEAST analyses using separately aligned coding regions with MAFFT (Katoh and Standley 2013), --*auto* setting and then concatenated sections. Using only genes with annotations in all species, we excluded all those with ambiguous annotations. Analyses were performed using a GTR+I+G substitution model (4 gamma categories), a strict molecular clock model, 200 millions generations (burn-in 10%) and *Trigonobalanus doichangensis* as outgroup. We checked chain convergence and Effective Sample Size (ESS) values > 200 using Tracer 1.6 (Rambaut et al. 2014).

### Comparison amongst oak species

We used the retrieved oak chloroplasts to estimate the genetic divergence amongst chloroplasts using MEGA6 (Tamura et al. 2013). We used an in-house script (available upon request) to evaluate the variability of aligned sequences amongst these five species, with a window length of 500 bp and a step size of 250 bp. Pair-to-pair comparisons were visualised using mVISTA (Frazer et al. 2004). We also assessed the number of private and shared SNPs amongst all the combination of species using a specifically developed script (available upon request). We applied Tajima’s relative rate test (Tajima 1993) on each species pair, using *Lithocarpus balansae* as outgroup to test the relative rate of evolution of *Q. xanthoclada* relative to other oak species.

## Results

### Chloroplast reconstruction

**Figure 1.**
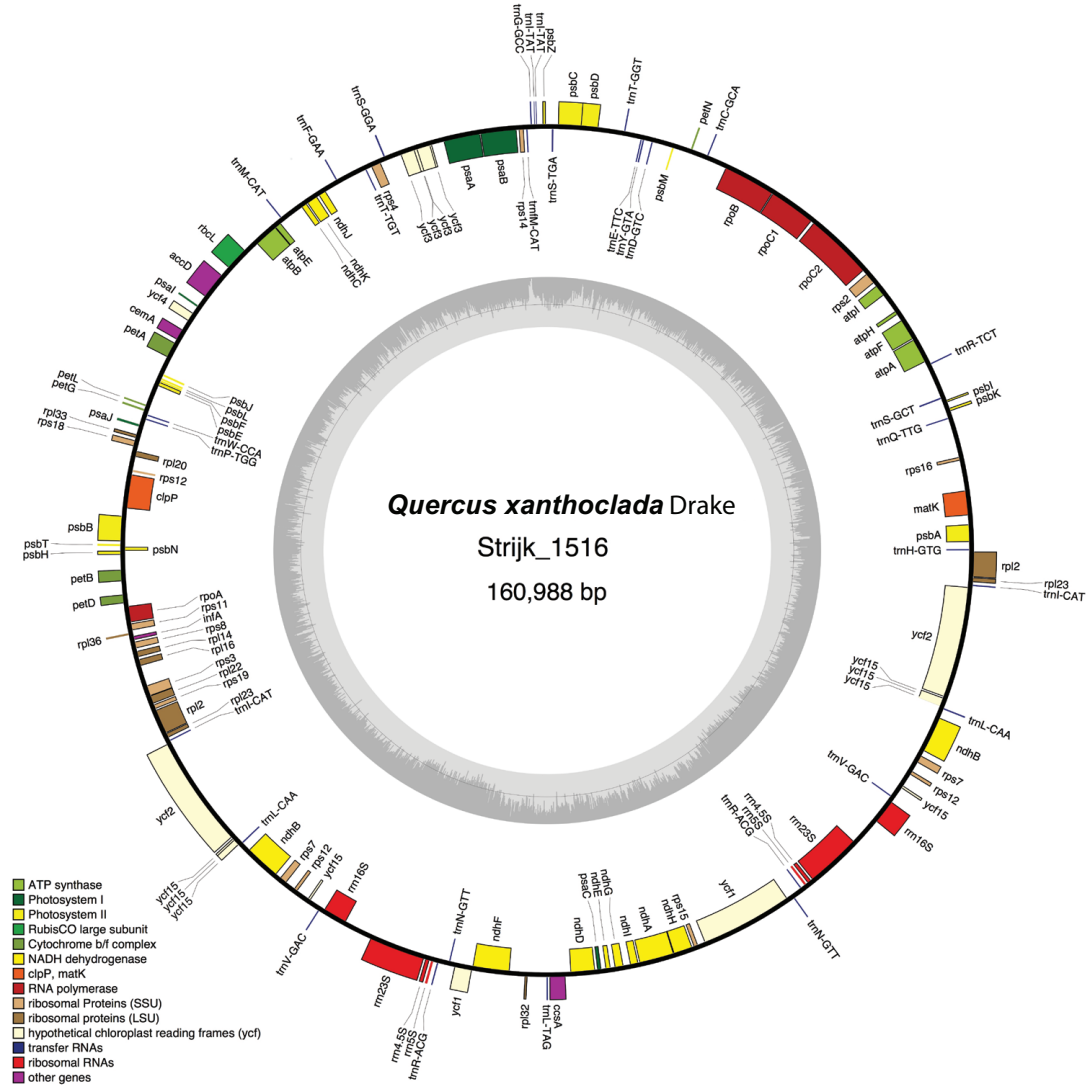
Circular gene map of the plastid genome of *Quercus xanthoclada*. Genes drawn within the circle are transcribed clockwise, while those drawn outside are transcribed counter clockwise. Genes are colour-coded according to their functional groups.

62,060 trimmed reads were mapped on the *Q. rubra* chloroplast sequence, for a total linear length of 162,328 bp. The main sequencing depth was 47.7 (min: 2; max: 90; S.D.: 12.5), covering 100% of the reference sequence (161,304 bp). The mean mapping confidence was 35.3, with 94.5% of the bases with mapping quality > Q20, and 89.0% with quality > Q30. The frequencies of each nucleotide were 31.1% (A), 18.7% (C), 18.0% (G) and 32.2% (T), with 439 positions unresolved (N). The properties of the *Quercus xanthoclada* plastid are shown in Table 1 and in Fig. 1. The chloroplast molecule was 160,988 bp long and was 163 bp, 67 bp and 573 bp longer than those of *Q. spinosa*, *Q. aliena* and *Q. aquifolioides*, respectively. In contrast, it was 316 bp shorter than the chloroplast sequence of *Q. rubra*. The overall GC content was 36.7%. The chloroplast structure conforms to the standard found in most angiosperms, with two IRs (25,840 bp) separated by a LSC (90,353 bp) and a SSC (18,955 bp). The plastid genome contains 129 genes, including 90 coding proteins, 31 tRNA and 8 rRNA (Fig. 1). Amongst them, six (*rpl2*, *ycf15* and *ndhB* duplicated in the IRs) and two included one or two introns, respectively.

**Table 1.** Characteristics of the complete chloroplasts used in this study, showing the length, the GC content of each regions and the number of coding, tRNA and rRNA loci. LSC: Large Single Copy region; SSC: Small Single Copy region; IR: Inverted Repeats. Data from Du et al. (2015) (*Q. spinosa*), Lu et al. (2016) (*Q. aliena*), Yin et al. (2018) (*Q. aquifolioides*, GenBank data), Alexander and Woeste (2014) (*Q. rubra*).

Species	length	LSC	SSC	IR	GC total	GC LSC	GC SSC	GC IR	coding	tRNA	rRNA
*Q. xanthoclada*	160,988	90,353	18,955	25,840	36.9	34.8	31.1	42.8	90	31	8
*Q. spinosa*	160,825	90,371	18,732	25,861	36.87	34.7	31.2	42.6	87	29	8
*Q. aliena*	160,921	90,258	18,980	25,841	36.9	34.8	31.3	42.7	89	39	8
*Q. aquifolioides*	160,415	89,856	18,935	25,812	37.0	36.6	31.2	42.8	78	29	8
*Q. rubra*	161,304	90,542	19,025	25,869	36.8	34.6	30.9	42.7	89	41	8

### Phylogenetic analysis and comparison amongst oak species and sections

**Figure 2.**
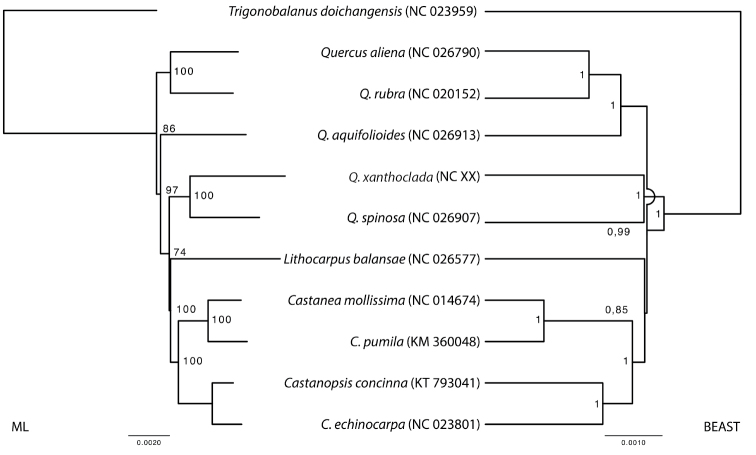
ML phylogenetic tree of the nine selected plastomes in GenBank, plus the plastome of *Quercus xanthoclada*. The tree is rooted with *Trigonobalanus doichangensis*. Bootstraps (1000 replicates) are shown at the nodes, values below 50% not shown. Scale in substitution per site.

The maximum likelihood (ML) tree of ten *Quercus* chloroplasts available in GenBank shows that *Q. xanthoclada* is closely related to *Q. spinosa* and to the clade formed by *Lithocarpus*, *Castanea* and *Castanopsis* (Fig. 2, left). In the Bayesian analysis, *Quercus* splits into two clades, one grouping *Q. xanthoclada* and *Q. spinosa*, the other containing the remaining species (*Q. aliena*, *Q. rubra*, *Q. aquifolioides*) (Fig. 2, right). The latter is the sister group of the clade comprising *Lithocarpus*, *Castanea* and *Castanopsis* species. Both trees are in general agreement, except for the placement of the clade containing *Q. xanthoclada*, that diverged after the other *Quercus* in the ML tree and before this split in the Bayesian tree. Nodal support was high in both trees for all groups, except the *Q. xanthoclada* – *Q. spinosa* clade (ML, 74% bootstrap support) and the *Lithocarpus* divergence (BEAST, 0.85 PP). In both trees, the internal branches are very short, indicating more mutational events occurred in each species than amongst genera or clades (with the notable exception of *Castanea*). Furthermore, considering only the *Quercus* species, *Q. xanthoclada* appears to have evolved significantly faster than the other oak species (Table 2).

**Table 2.** Results of the Tajima relative rate test. ***X***^2^ test statistic value indicated, p-value in parenthesis. p-values < 0.01 in bold. For significant rate heterogeneity comparisons, the species with slower evolutionary rate is indicated: Qsp: *Q. spinosa*, Qal: *Q. aliena*, Qaq: *Q. aquifolioides*, Qru: *Q. rubra*. For clarity, p-values lower than 0.005 are not indicated.

Species	*Q. xanthoclada*	*Q. spinosa*	*Q. aliena*	*Q. aquifolioides*	*Q. rubra*
*Q. xanthoclada*	/	17.37 – Qsp	30.35 – Qal	10.37 – Qaq	32.64 – Qru
*Q. spinosa*		/	4.17 (0.04) – Qal	0.02 (0.89)	3.47 (0.06)
*Q. aliena*			/	4.99 (0.03)-Qal	0.06 (0.81)
*Q. aquifolioides*				/	3.30 (0.07)
*Q. rubra*					/

**Figure 3.**
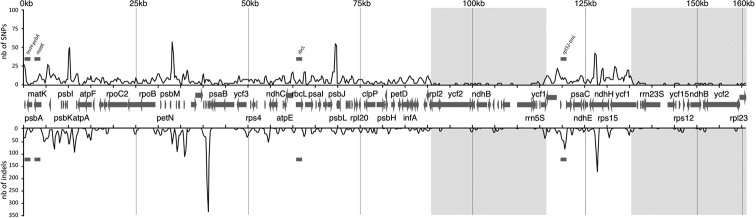
Sliding window analysis of the whole plastomes of five oak species. (window length: 500 bp, step size: 250 bp). X-axis: position of the mid-point of the window, Y-axis: number of SNPs (solid line) and indels (dashed line) positions of each window in bp. Coding regions and directions of transcription are indicated by arrows, inverted repeats by grey areas. Putative barcode loci are highlighted for SNPs and indels. For readability, only a few major genes are indicated.

All five oak species exhibited high overall similarities (99.4–99.6%) (Table 3), but the 3766 SNPs detected were distributed unevenly across and within the plastid alignment (Fig. 3). Unsurprisingly, most of the variability amongst species was concentrated in the intergenic spacers and the gene introns. Four regions were especially SNPs-rich: the 5’end of the *trnS-GCT*-*trnR-TCT* spacer (49 SNPs, 750 bp), the *psbM*-*trnD-GTC* spacer (50 SNPs, 750 bp), the *petA*-*psbJ* spacer (55 SNPs, 750 bp) and the 3’end of *ndhA* (41 SNPs, 750 bp). Two regions exhibited a dramatic increase in the number of indel positions, namely the *psbZ*-*rps14* interval (comprising *trnI-TAT*, *trnfM-CAT* and *trnG-GCC*, 348 indel positions per kb) and the 3’ end of *ndhA* (188 indel position per kb). However, the high number of indel positions in the first of these intervals actually represents regions where more than 70% of the positions were deleted in at least one species (results not shown). The less variable regions were both the IRs, with almost no SNPs or indels.

**Table 3.** Estimates of p-distance amongst oak species. The number of base differences per site is shown. All positions containing gaps and missing data were eliminated. There were a total of 158480 positions in the final dataset.

Species	*Q. xanthoclada*	*Q. spinosa*	*Q. aliena*	*Q. aquifolioides*	*Q. rubra*
*Q. xanthoclada*	/	0.005	0.006	0.006	0.006
*Q. spinosa*		/	0.005	0.005	0.005
*Q. aliena*			/	0.005	0.004
*Q. aquifolioides*				/	0.005
*Q. rubra*					/

**Figure 4.**
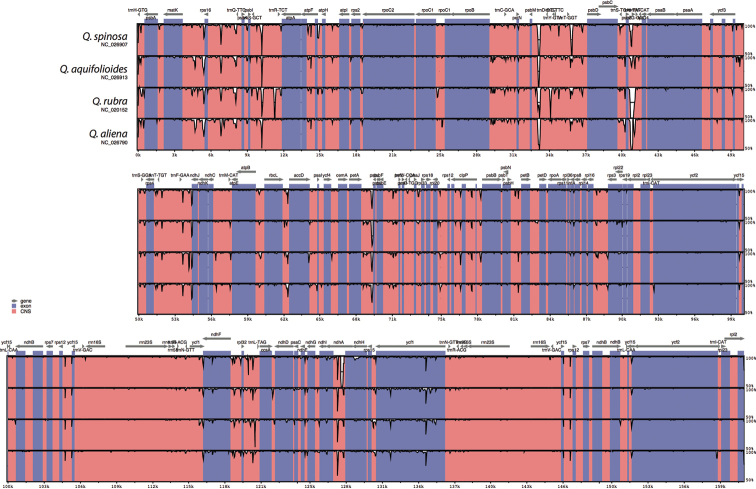
mVISTA percent identity plot comparing the four *Quercus* chloroplast genomes with *Q. xanthoclada* as a reference. Vertical scale indicates the percentage of identity ranging from 50% to 100%. Coding regions are in blue and non-coding regions are in pink.

However, this overall pattern varied when considering each species pair (Fig. 4). Despite most of the overall divergent regions being equally divergent in all species, several variable regions appeared to be more species specific: *atpF*/*atpH*, the 5’end of *trnT*-*GGT* and the 5’end of the *ndhA* intron in *Q. spinosa*, the 3’end of *trnS*-*GCT*/*trnR*-*TCT*, the intron of *rpoC1* and the *trnD*-*GTC*/*trnY-GTA* spacer in *Q. rubra*. In addition, both the *psbZ*/*trnI-TAT* (comprising *trnG-GCC*) and the *rps15*/*ycf1* spacers were more divergent from *Q. xanthoclada* in *Q. rubra* and *Q. aliena* than in *Q. spinosa* and *Q. aquifolioides*. Interestingly, the divergent portions of the *rpl32*/*trnL* spacer were different for each species. No notable divergent regions were found in the two IRs in all species pairs.

## Discussion

The genome-skimming approach is now widely used to reconstruct chloroplasts in angiosperms (Straub et al. 2012; Bock et al. 2014; Malé et al. 2014; Weitemier et al. 2014; Mandel et al. 2015) and an increasing number of Fagales are available (Alexander and Woeste 2014; El Mujtar et al. 2014; Hinsinger and Strijk 2015). Since the first Fagaceae (*C. concinna*; Hinsinger and Strijk 2015), the genome skimming approach has been proven to be useful in many non-model organisms (Kane et al. 2012; Nikiforova et al. 2013; Curci et al. 2016; Yu et al. 2018), despite a few disadvantages (Straub et al. 2012; Du et al. 2015), as genome skimming is a PCR-free method and a higher amount of starting material is required. Moreover, best results require high quality DNA (i.e high molecular weight DNA, usually > 10 kb, with little degradation), which is incompatible with large-scale (i.e. phylogenetic or population genomic) sampling. Nonetheless, the protocols can easily be adapted (e.g. using specific library construction kits (Zedane et al. 2016) or skipping the shearing step in degraded samples like herbarium or archaeological specimens (Särkinen et al. 2012; Staats et al. 2013). Several examples showcasing the strength of these approaches have been published. For example, Zedane et al. (2016) explored the evolutionary relationships of *Hesperelaea*, an extinct Oleaceae, by reconstructing the plastome of a century-old herbarium specimen and Renner et al. (2019) identified 3,500 years old archaeological remains as watermelon, based on their plastome sequence. With the constant progress in sequencing technology and library construction and the development of genomic resources for oaks (Kremer et al. 2012; Hipp et al. 2014), we can expect that these limitations will soon be overcome.

In addition to the complete chloroplast sequence, the genome skimming can also be use to retrieve nuclear regions found in high copy number in the genome, such as the nuclear ribosomal cistrons (NRC) (Straub et al. 2012; Ripma et al. 2014; Weitemier et al. 2014). To evaluate the relative variability of the complete chloroplast sequence versus the nuclear ribosomal cistrons, we attempted to retrieve the available raw data for the considered oak species from the Short Reads Archive (SRA). Despite the stringent chloroplast purification protocol described in Du et al. (2015), *Q. spinosa* was the only species where we were able to retrieve the NRC, following the same protocol as described above, mapping the trimmed reads against the nuclear internal transcribed spacer of *Q. robur* (FM244246) (results not shown).

In our study, the plastome of *Q. rubra* was the largest in size in oaks (161,304 bp), but in other studies, it was described as the second smallest in oaks (Alexander and Woeste 2014). Given the species diversity in oaks and with an increasing number of plastomes available, this view is likely to change in the near future.

*Quercus* is widespread throughout the whole of the northern hemisphere, but in our study, three of the four available species came from Asia. To fully capture the chloroplast sequence diversity on a global scale, future inclusion of and genomic comparison with American and European *Quercus* is needed. Although species in this study are members of different recognised sections and subgenera, it is likely that they represent only a fraction of the total diversity within each of these groups and inclusion of additional members will reveal more about the extent and distribution of plastome diversity on various taxonomic and global scales.

Interestingly, one of the regions showing a relatively high level of variation amongst oak species is the *rpl32*-*trnL* spacer. As the observed variations are located in different portions of the region, it is likely that these SNPs and indels represent section specific diagnostic regions. Indeed, *rpl32*-*trnL* has been hypothesised as a DNA-barcode locus in several groups (Shaw et al. 2007; Arca et al. 2012). Other proposed DNA-barcode loci showed different levels of variation, *rbcL* being the least variable, with no indels and very few SNPs, as previously demonstrated in several woody plants (Chase et al. 2005; Roy et al. 2010; Arca et al. 2012; Clement and Donoghue 2012). In contrast, *trnH*-*psbA* has been shown to be highly variable (Piredda et al. 2010; Arca et al. 2012; Clement and Donoghue 2012; Hinsinger et al. 2013), mostly for SNPs, while *rpl32*-*trnL* was more variable for indels. Briefly, the proposed standard DNA-barcode loci (namely *rbcL* and *matK* (Hollingsworth et al. 2009)) would likely fail in oaks, as demonstrated for certain subclades or specific geographic areas (Piredda et al. 2010; Simeone et al. 2013). However, not only the divergence of putative barcode loci amongst distantly related species has to be assessed, but also between closely related species, as well as amongst different populations. We highlight that *rpl32*-*trnL*, as well as trnH-*psbA*, should be assessed for DNA-barcoding purpose in oaks, in addition to a thorough re-assessment and estimation of the usefulness of the proposed standard loci *rbcL* and *matK*.

**Figure 5.**
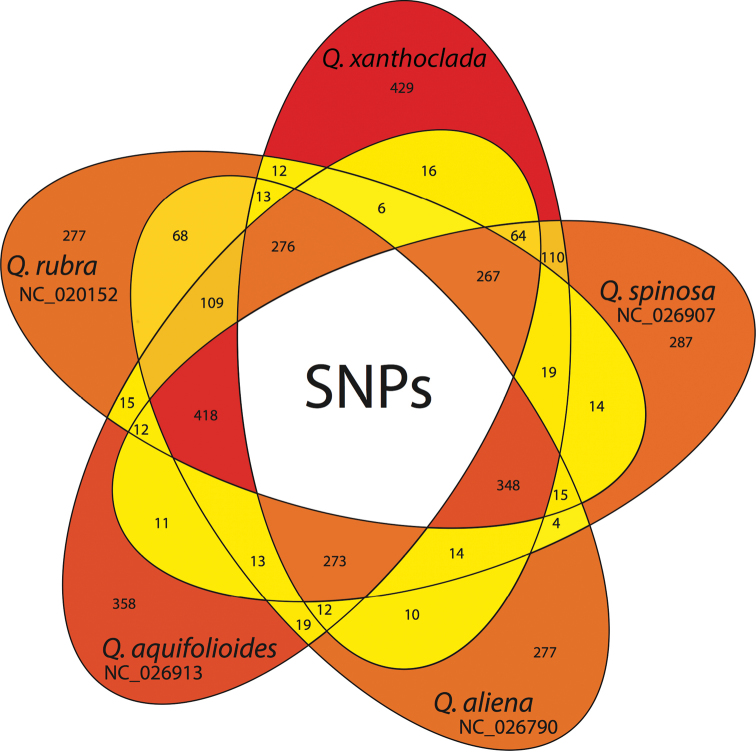
Venn diagram showing the private and shared SNPs amongst the five oak species. Each area is coloured according to the relative number of shared SNPs in this area.

Most of the SNPs were either specific to one species or shared by four species and only a few shared by only two species (Fig. 5). *Q. xanthoclada* exhibited the highest number of species specific SNPs (429), whereas *Q. aliena* and *Q. rubra* segregated the lowest number of SNPs (277). Accordingly, *Q. xanthoclada* was excluded from the four species group that shared the highest number of SNPs (418), followed by *Q. aquifolioides* (348).

Although these results, in combination with those obtained in Tajima’s relative rate test, seem to suggest a relative distinction of the *Cyclobalanopsis* species and their previous separation as a separate subgenus in the genus *Quercus*, this is not corroborated by any other data in our study. Neither the complete chloroplast phylogeny, nor previous studies based on nuclear and chloroplast loci (Manos et al. 2001; Oh and Manos 2008), justify this separation of *Q. xanthoclada* (and other taxa under the generic or subgeneric header of “*Cyclobalanopsis*”) from other oaks.

Our work will allow for the development of new loci to be used in comparative phylogeography of the different section and subgenera (i.e. primers that are easily transferable amongst the different sections that can occur in sympatry), as well as open up new perspectives for conservation, management and the use of DNA fingerprinting to aid tracking of wood products from subtropical and tropical Asian oak species.

## References

[B1] Alexander LW Woeste KE (2014) Pyrosequencing of the northern red oak (*Quercus rubra* L.) chloroplast genome reveals high quality polymorphisms for population management. Tree Genetics & Genomes 10(4): 803–812. 10.1007/s11295-013-0681-1

[B2] Arca M Hinsinger DD Cruaud C Tillier A Bousquet J Frascaria-Lacoste N (2012) Deciduous trees and the application of universal dna barcodes: A case study on the circumpolar fraxinus. PLoS One 7(3): e34089. 10.1371/journal.pone.0034089 PMC3313964 22479532

[B3] Bakker FT Lei D Yu J Mohammadin S Wei Z van de Kerke S Gravendeel B Nieuwenhuis M Staats M Alquezar-Planas DE Holmer R (2016) Herbarium genomics: Plastome sequence assembly from a range of herbarium specimens using an Iterative Organelle Genome Assembly pipeline. Biological Journal of the Linnean Society. Linnean Society of London 117(1): 33–43. 10.1111/bij.12642

[B4] Bock DG Kane NC Ebert DP Rieseberg LH (2014) Genome skimming reveals the origin of the Jerusalem Artichoke tuber crop species: Neither from Jerusalem nor an artichoke. The New Phytologist 201(3): 1021–1030. 10.1111/nph.12560 24245977

[B5] Botero-Castro F Delsuc F Douzery EJP (2016) Thrice better than once: Quality control guidelines to validate new mitogenomes. Mitochondrial DNA 27(1): 449–454. 10.1111/nph.12560 24708133

[B6] Bouckaert R Heled J Kühnert D Vaughan T Wu CH Xie D Suchard MA Rambaut A Drummond AJ (2014) BEAST 2: A Software Platform for Bayesian Evolutionary Analysis. PLoS Computational Biology 10(4): e1003537. 10.1371/journal.pcbi.1003537 PMC3985171 24722319

[B7] Camus A (1936–1954) Les chenes monographie du genre *Quercus* (et *Lithocarpus*). Encyclopédie économique de sylviculture 6: 8.

[B8] Chase MW Salamin N Wilkinson M Dunwell JM Kesanakurthi RP Haidar N Savolainen V (2005) Land plants and DNA barcodes: Short-term and long-term goals. Philosophical Transactions of the Royal Society B 360: 1889–1895. 10.1098/rstb.2005.1720 PMC1609218 16214746

[B9] Clement WL Donoghue MJ (2012) Barcoding success as a function of phylogenetic relatedness in *Viburnum*, a clade of woody angiosperms. BMC Evolutionary Biology 12(1): 73 10.1186/1471-2148-12-73 22646220 PMC3433329

[B10] Curci PL De Paola D Sonnante G (2016) Development of chloroplast genomic resources for *Cynara*. Molecular Ecology Resources 16(2): 562–573. 10.1111/1755-0998.12457 26354522

[B11] Cvetković T Hinsinger DD Strijk JS (2017) The first complete chloroplast sequence of a major tropical timber tree in the Meranti family: *Vatica odorata* (Dipterocarpaceae). Mitochondrial DNA. Part B, Resources 2(1): 52–53. 10.1080/23802359.2016.1275837 PMC7800909 33473714

[B12] Cvetković T Hinsinger DD Strijk JS (2019) Living on the edge: Exploring genomic diver-sity in Chinese Dipterocarpaceae using NGS data. Scientific Reports 9: 11639. 10.1038/s41598-019-48240-y PMC6690942 31406227

[B13] Denk T Grimm GW (2009) Significance of pollen characteristics for infrageneric classification and phylogeny in *Quercus* (Fagaceae). International Journal of Plant Sciences 170(7): 926–940. 10.1086/600134

[B14] Denk T Grimm GW Manos PS Deng M Hipp AL (2017) An updated infrageneric classification of the oaks: Review of previous taxonomic schemes and synthesis of evolutionary patterns – oaks physiological ecology. Exploring the functional diversity of genus *Quercus* L. Tree Physiology 7: 13–38. 10.1007/978-3-319-69099-5_2

[B15] Du FK Lang T Lu S Wang Y Li J Yin K (2015) An improved method for chloroplast genome sequencing in non-model forest tree species. Tree Genetics & Genomes 11(6): 114 10.1007/s11295-015-0942-2

[B16] El Mujtar VA Gallo LA Lang T Garnier-Géré P (2014) Development of genomic resources for *Nothofagus* species using next-generation sequencing data. Molecular Ecology Resources 14(6): 1281–1295. 10.1111/1755-0998.12276 24813056

[B17] Frazer KA Pachter L Poliakov A Rubin EM Dubchak I (2004) VISTA: Computational tools for comparative genomics. Nucleic Acids Research 32(Web Server): W273–W279. 10.1093/nar/gkh458 PMC441596 15215394

[B18] Guindon S Delsuc F Dufayard JF Gascuel O (2009) Estimating maximum likelihood phylogenies with PhyML. Methods in Molecular Biology (Clifton, N.J.) 537: 113–137. 10.1007/978-1-59745-251-9_6 19378142

[B19] Healey A Furtado A Cooper T Henry RJ (2014) Protocol: A simple method for extracting next-generation sequencing quality genomic DNA from recalcitrant plant species. Plant Methods 10(1): 1 10.1186/1746-4811-10-21 25053969 PMC4105509

[B20] Hinsinger DD Strijk JS (2015) Complete chloroplast genome sequence of *Castanopsis concinna* (Fagaceae), a threatened species from Hong Kong and South-Eastern China. Mitochondrial DNA. Part A, DNA Mapping, Sequencing, and Analysis 28(1): 65–66. 10.3109/19401736.2015.1110800 26678387

[B21] Hinsinger DD Basak J Gaudeul M Cruaud C Bertolino P Frascaria-Lacoste N Bousquet J (2013) The phylogeny and biogeographic history of ashes (*Fraxinus*, Oleaceae) highlight the roles of migration and vicariance in the diversification of temperate trees. PLoS One 8(11): e80431. 10.1371/journal.pone.0080431 PMC3837005 24278282

[B22] Hipp AL Eaton DAR Cavender-Bares J Fitzek E Nipper R Manos PS (2014) A framework phylogeny of the American oak clade based on sequenced RAD data. PLoS One 9(4): e93975. 10.1371/journal.pone.0093975 PMC3976371 24705617

[B23] Hollingsworth PM Forrest LL Spouge JL Hajibabaei M Ratnasingham S van der Bank M Chase MW Cowan RS Erickson DL Fazekas AJ Graham SW James KE Kim K-J Kress WJ Schneider H van AlphenStahl J Barrett SCH van den Berg C Bogarin D Burgess KS Cameron KM Carine M Chacon J Clark A Clarkson JJ Conrad F Devey DS Ford CS Hedderson TAJ Hollingsworth ML Husband BC Kelly LJ Kesanakurti PR Kim JS Kim Y-D Lahaye R Lee H-L Long DG Madrinan S Maurin O Meusnier I Newmaster SG Park C-W Percy DM Petersen G Richardson JE Salazar GA Savolainen V Seberg O Wilkinson MJ Yi D-K Little DP CBOL plant Working Group (2009) A DNA barcode for land plants. Proceedings of the National Academy of Sciences of the United States of America 106(31): 12794–12797. 10.1073/pnas.0905845106 19666622 PMC2722355

[B24] Huang C Chang Y Hsu Y Jen H (1998) Flora Reipublicae Popularis Sinicae. In: Chun W Huang C (Eds) Republ.Popularis Sin, 1–332.

[B25] Huang C Zhang Y Bartholomew B (2009) Fagaceae. In: Wu Z-Y Raven P (Eds) Flora of China 4.Cycadaceae through Fagaceae. Science Press and Missouri Botanical Garden Press, Beijing & St. Louis, 314–400.

[B26] Jansen RK Ruhlman TA (2012) Plastid Genomes of Seed Plants. Springer, Dordrecht, 103–126. 10.1007/978-94-007-2920-9_5

[B27] Kane N Sveinsson S Dempewolf H Yang JY Zhang D Engels JMM Cronk Q (2012) Ultra-barcoding in cacao (*Theobroma* spp.; Malvaceae) using whole chloroplast genomes and nuclear ribosomal DNA. American Journal of Botany 99(2): 320–329. 10.3732/ajb.1100570 22301895

[B28] Katoh K Standley DM (2013) MAFFT multiple sequence alignment software version 7: Improvements in performance and usability. Molecular Biology and Evolution 30(4): 772–780. 10.1093/molbev/mst010 23329690 PMC3603318

[B29] Kremer A Abbott AG Carlson JE Manos PS Plomion C Sisco P Staton ME Ueno S Vendramin GG (2012) Genomics of Fagaceae. Tree Genetics & Genomes 8(3): 583–610. 10.1007/s11295-012-0498-3

[B30] Linnaeus C (1753) Species plantarum: exhibentes plantas rite cognitas ad genera relatas cum differentiis specificis, nominibus trivialibus, synonymis selectis, locis natalibus secundum systema sexuale digestas. 10.5962/bhl.title.669

[B31] Liu C Shi L Zhu Y Chen H Zhang J Lin X Guan X (2012) CpGAVAS, an integrated web server for the annotation, visualization, analysis, and GenBank submission of completely sequenced chloroplast genome sequences. BMC Genomics 13(1): 715 10.1186/1471-2164-13-715 23256920 PMC3543216

[B32] Lohse M Drechsel O Bock R (2007) OrganellarGenomeDRAW (OGDRAW) – a tool for easy generation of high-quality custom graphical maps of plastid and mitochondrial genomes. Current Genetics: 52. 10.1007/s00294-007-0161-y 17957369

[B33] Lu S Hou M Du FK Li J Yin K (2016) Complete chloroplast genome of the Oriental white oak: *Quercus aliena* Blume. Mitochondrial DNA 27: 2802–2804. 10.3109/19401736.2015.1053074 26114324

[B34] Luo Y Zhou ZK (2002) Leaf architecture in Quercus subgenus Cyclobalanopsis (Fagaceae) from China. Botanical Journal of the Linnean Society 140(3): 283–295. 10.1046/j.1095-8339.2002.00097.x

[B35] Malé PJG Bardon L Besnard G Coissac E Delsuc F Engel J Lhuillier E Scotti-Saintagne C Tinaut A Chave J (2014) Genome skimming by shotgun sequencing helps resolve the phylogeny of a pantropical tree family. Molecular Ecology Resources 14: 966–975. 10.1111/1755-0998.12246 24606032

[B36] Mandel JR Dikow RB Funk VA (2015) Using phylogenomics to resolve mega-families: An example from Compositae. Journal of Systematics and Evolution 53(5): 391–402. 10.1111/jse.12167

[B37] Manos PS Zhou Z Cannon CH (2001) Systematics of Fagaceae: Phylogenetic tests of reproductive trait evolution. International Journal of Plant Sciences 162(6): 1361–1379. 10.1086/322949

[B38] Nikiforova SV Cavalieri D Velasco R Goremykin V (2013) Phylogenetic analysis of 47 chloroplast genomes clarifies the contribution of wild species to the domesticated apple maternal line. Molecular Biology and Evolution 30(8): 1751–1760. 10.1093/molbev/mst092 23676769

[B39] Nixon KC (2006) Global and neotropical distribution and diversity of oak (genus *Quercus*) and oak forests, ecology and conservation of neotropical montane oak forests. Springer, 3–13. 10.1007/3-540-28909-7_1

[B40] Oh SH Manos PS (2008) Molecular phylogenetics and cupule evolution in Fagaceae as inferred from nuclear CRABS CLAW sequences. Taxon 57: 434–451.

[B41] Pham KK Hipp AL Manos PS Cronn RC (2017) A time and a place for everything: Phylogenetic history and geography as joint predictors of oak plastome phylogeny. Genome 60(9): 1–13. 10.1139/gen-2016-0191 28445658

[B42] Phengklai C (2008) Flora of Thailand: Fagaceae. In: Santisuk T, Larsen K, Nielsen I, Chayamarit K, Phengkhlai C, Pedersen H, Parnell J, Middleton D, Newman M, Simpson DA, van Welzen PC, Hul S, Kato M (Eds) The Forest Herbarium, National Parks, Wildlife and Conservation Department, Bangkok.

[B43] Piredda R Simeone MC Attimonelli M Bellarosa R Schirone B (2010) Prospects of barcoding the Italian wild dendroflora: Oaks reveal severe limitations to tracking species identity. Molecular Ecology Resources 11(1): 72–83. 10.1111/j.1755-0998.2010.02900.x 21429102

[B44] Pulido MT Cavelier J Cortés SP (2006) Structure and composition of Colombian montane oak forests, ecology and conservation of neotropical montane oak forests. Springer, 141–151. 10.1007/3-540-28909-7_11

[B45] Rambaut A Drummond AJ Suchard M (2014) Tracer v.1.6. http://tree.bio.ed.ac.uk/software/tracer/ss

[B46] Renner SS Pérez-Escobar OA Silber MV Nesbitt M Preick M Hofreiter M Chomicki G (2019) A 3500-year-old leaf from a Pharaonic tomb reveals that New Kingdom Egyptians were cultivating domesticated watermelon. bioRxiv 642785. 10.1101/642785

[B47] Ripma LA Simpson MG Hasenstab-Lehman K (2014) Geneious! Simplified genome skimming methods for phylogenetic systematic studies: A case study in *Oreocarya* (Boraginaceae). Applications in Plant Sciences 2(12): 1400062 10.3732/apps.1400062 PMC4259456 25506521

[B48] Roy S Tyagi A Shukla V Kumar A Singh UM Chaudhary LB Datt B Bag SK Singh PK Nair NK Husain T Tuli R (2010) Universal plant DNA barcode loci may not work in complex groups: A case study with Indian berberis species. PLoS One 5(10): e13674. 10.1371/journal.pone.0013674 PMC2965122 21060687

[B49] Särkinen T Staats M Richardson JE Cowan RS Bakker FT (2012) How to open the treasure chest? Optimising DNA extraction from herbarium specimens. PLoS One 7(8): e43808. 10.1371/journal.pone.0043808 PMC3429509 22952770

[B50] Shaw J Lickey EB Schilling EE Small RL (2007) Comparison of whole chloroplast genome sequences to choose noncoding regions for phylogenetic studies in angiosperms: The tortoise and the hare III. American Journal of Botany 94(3): 275–288. 10.3732/ajb.94.3.275 21636401

[B51] Simeone MC Piredda R Papini A Vessella F Schirone B (2013) Application of plastid and nuclear markers to DNA barcoding of Euro-Mediterranean oaks (*Quercus*, Fagaceae): Problems, prospects and phylogenetic implications. Botanical Journal of the Linnean Society 172(4): 478–499. 10.1111/boj.12059

[B52] Soepadmo E Steenis CGGJ (1972) Fagaceae. Flora Malesiana-Series 1. Spermatophyta 7: 265–403.

[B53] Staats M Erkens RHJ van de Vossenberg B Wieringa JJ Kraaijeveld K Stielow B Geml J Richardson JE Bakker FT (2013) Genomic treasure troves: Complete genome sequencing of herbarium and insect museum specimens. PLoS One 8(7): e69189. 10.1371/journal.pone.0069189 PMC3726723 23922691

[B54] Straub SCK Parks M Weitemier K Fishbein M Cronn RC Liston A (2012) Navigating the tip of the genomic iceberg: Next-generation sequencing for plant systematics. American Journal of Botany 99(2): 349–364. 10.3732/ajb.1100335 22174336

[B55] Strijk JS (2019) The Complete Database for Information on the Evolutionary History, Diversity, Identification and Conservation of Over 700 Species of Asian Trees. https://www.asianfagaceae.com [Retrieved on August 4 2019]

[B56] Tajima F (1993) Simple methods for testing the molecular evolutionary clock hypothesis. Genetics 135: 599–607. 8244016 10.1093/genetics/135.2.599 PMC1205659

[B57] Tamura K Stecher G Peterson D Filipski A Kumar S (2013) MEGA6: Molecular Evolutionary Genetics Analysis version 6.0. Molecular Biology and Evolution 30(12): 2725–2729. 10.1093/molbev/mst197 24132122 PMC3840312

[B58] Weitemier K Straub SCK Cronn RC Fishbein M Schmickl R McDonnell A Liston A (2014) Hyb-Seq: Combining Target enrichment and genome skimming for plant phylogenomics. Applications in Plant Sciences 2(9): 1400042 10.3732/apps.1400042 PMC4162667 25225629

[B59] Yin K Zhang Y Li Y Du FK (2018) Different natural selection pressures on the *atpF* gene in evergreen sclerophyllous and deciduous oak species: evidence from comparative analysis of the complete chloroplast genome of *Quercus aquifolioides* with other oak species. International Journal of Molecular Sciences 19(4): 1042 10.3390/ijms19041042 PMC5979438 29601535

[B60] Yu T Hinsinger DD Strijk JS Wee AKS (2018) The first complete chloroplast genome of a major mangrove species *Sonneratia alba* Sm. and its implications on conservation efforts. Mitochondrial DNA. Part B, Resources 3(2): 500–502. 10.1080/23802359.2018.1463828 PMC7799946 33474220

[B61] Zedane L Hong-Wa C Murienne J Jeziorski C Baldwin BG Besnard G (2016) Museomics illuminate the history of an extinct, paleoendemic plant lineage (Hesperelaea, Oleaceae) known from an 1875 collection from Guadalupe Island, Mexico. Biological Journal of the Linnean Society. Linnean Society of London 117(1): 44–57. 10.1111/bij.12509

